# New Data on Ixodid Ticks and Their Infection with *Borrelia* and *Coxiella burnetii* in Vietnam

**DOI:** 10.3390/tropicalmed11050126

**Published:** 2026-05-06

**Authors:** Nguyen Van Hiep, Tatiana A. Bondarenko, Le Thi Lan Anh, Olga A. Stukolova, Luong Thi Mo, Kseniia A. Sycheva, Vien Chinh Chien, Alex L. Viskontene, Nguyen Thi Dung, Dmitriy V. Dubrovskiy, Truong Xuan Toan, Marina I. Sokolova, Truong Thi Ngan, Irina P. Lisyukova, Daria D. Skripnichenko, Viktoria P. Bulanenko, Yulia V. Fedakova, Vasily G. Akimkin, Marat T. Makenov

**Affiliations:** 1 Southern Branch of Joint Vietnam-Russia Tropical Science and Technology Research Center, Vuon Lai Ward, Ho Chi Minh City 740500, Vietnam; 2 Central Research Institute of Epidemiology, Moscow 111123, Russia; 3 Institute of Tropical Medicine of Joint Vietnam-Russia Tropical Science and Technology Research Center, Nghia Do, Hanoi 122100, Vietnam; 4 Tay Nguyen Institute of Hygiene and Epidemiology, Tan An Ward, Buon Ma Thuot City 631554, Vietnam

**Keywords:** Ixodidae, Iixodid ticks, Amblyomma, tick-borne diseases, *Borrelia theileri*, Bovine borreliosis, *Coxiella burnetii*, Q fever, Zoonoses

## Abstract

The distribution of medically significant ticks in Vietnam requires ongoing monitoring. This study presents data on tick distribution and molecular screening for *Borrelia* spp. and *Coxiella burnetii* DNA. Ticks were collected from domestic animals and vegetation in four provinces over the period of 2024–2025. Species identification was performed based on morphology and confirmed by sequencing mitochondrial COI and 16S rRNA genes. A total of 2347 ticks were collected, representing eight species from the genera *Haemaphysalis*, *Rhipicephalus*, and *Amblyomma*. The study provides new distribution records for *H. bispinosa*, *H. cornigera*, *A. integrum*, and several rarely reported species (*H. lagrangei*, *H. hystricus*, and *H. wellingtoni*). PCR screening revealed a relatively high detection rate of *Borrelia* DNA in *H. cornigera* from Cao Bang province. Sequencing identified the pathogen as *B. theileri*, the agent of bovine borreliosis. *Borrelia theileri* was also detected in *R. microplus* in other regions, indicating wider circulation. PCR screening for *Coxiella burnetii* was positive for 13 ticks from cattle in Cao Bang province. To rule out false-positive results due to detection of DNA from *Coxiella*-like endosymbionts, we sequenced a fragment of the IS1111 element for three positive samples. The sequences confirmed that the DNA belongs to bacteria of the genus *Coxiella*, but the data do not allow confident assignment to *C. burnetii* at the species level. These positive ticks originated from eight neighboring households, suggesting a potential localized focus that requires further assessment in livestock and humans to determine the epidemiological significance. This research enhances the understanding of Vietnam’s tick fauna and associated pathogens of medical and veterinary importance.

## 1. Introduction

Ixodid ticks represent a group of great importance in public and veterinary health, serving as vectors for a diverse list of viral, bacterial, and protozoan pathogens. The distribution of tick-borne infections is largely constrained by the ecological ranges of their competent vectors. However, ongoing climate change and intensified anthropogenic activities are progressively altering these historical biogeographical boundaries. Consequently, tick species are increasingly being documented in non-endemic regions [1,2,3,4,5,6,7], facilitating the emergence of novel pathogens in immunologically naive human and animal populations. Therefore, establishing baseline data on indigenous tick fauna and the pathogens they harbor is essential for preparedness and effective response to potential outbreaks.

The tick fauna of Vietnam has been extensively studied, with research continuing to the present day [8,9,10]. Previous studies documented 59 tick species across seven genera, with the genus *Haemaphysalis* identified as the most species-rich [11]. More recent investigations conducted in 2024-2025 have expanded this number to 66 species, further refining our understanding of their provincial distribution and host preferences [11,12].

In parallel with these acarological surveys, significant efforts have been directed toward characterizing the spectrum of tick-borne pathogens in the region. The established list of circulating agents in Vietnam includes spotted fever group rickettsiae (*Rickettsia japonica*, *R. felis*), bacterial pathogens of the genera *Anaplasma* (*A. phagocytophilum*, *A. platys*), and spirochetes from *Borrelia* (*B. valaisiana*, *B. miyamotoi*) [10,13,14]. A notable public health threat emerged in 2017 with the first reported human cases of Severe Fever with Thrombocytopenia Syndrome (SFTS), caused by a phlebovirus of the family *Phenuiviridae*, in Thua Thien Hue province [15]. Given the severe morbidity associated with this tick-transmitted virus [16,17], it is crucial to investigate ticks in this and adjacent provinces to delineate the natural foci of SFTSV transmission.

The application of advanced molecular tools, such as metagenomic sequencing, in neighboring countries has proven highly effective. This approach has not only confirmed the presence of endemic pathogens [18,19,20,21,22,23,24] but has also led to the discovery of a multitude of novel viruses, including Yezo virus, Songling virus, Beiji virus, Guertu virus, and Jingmen tick virus [25,26,27,28,29]. Considering that competent vectors for these pathogens are present within Vietnam, there is a clear research gap regarding the diversity and distribution of tick-borne pathogens in the country. In particular, there is limited data on the prevalence of tick-borne infections such as borreliae in Vietnam, including their species identification and assessment of their medical and veterinary significance [14]. Similarly, data on *Coxiella burnetii*, the causative agent of Q fever, remain limited in Vietnam [18]. Although serological studies from Southeast Asia confirm its circulation in livestock and humans, including a recent human case reported in the country [30], its prevalence has yet to be systematically assessed.

Therefore, the present study aims to address this gap by providing updated data on the distribution of ixodid tick species across Vietnam and characterizing the infection rate of bacterial tick-borne pathogens within their populations.

## 2. Materials and Methods

### 2.1. Tick Sampling and Preparation

Tick collections were conducted in selected provinces of northern and southern Vietnam. The sampling sites included Na Hau National Park and adjacent communes in Yen Bai Province, and Phia Oac-Phia Den National Park and adjacent communes in Cao Bang Province, with field work undertaken between November and December 2024. Further collections were carried out in Cat Tien National Park and adjacent communes in Dong Nai Province, and Chu Mom Ray National Park and adjacent communes in Quang Ngai Province during July and August 2025. 

Ticks were collected from domestic animals, specifically cows, buffaloes, and dogs. This involved visiting households and, upon obtaining owner consent, examining animals and collecting ticks into sterile vials. All ticks from a single animal were placed into one vial. The vials were labeled with the collection date, host animal, and location. Additionally, questing ticks were collected from vegetation using the flagging method. For this, surveyors traversed a 1–3 km transect through the most typical biotopes, dragging a flag and collecting any adhering ticks.

In the laboratory, ticks were morphologically identified to species level using standard taxonomic keys [18,30,31]. Following identification, ticks were pooled, with each pool containing one to three individuals, with the exception of flag-collected larvae, which were pooled in groups of 15 per pool. Pools were assembled based on tick species, life stage/sex, and host animal or collection location for flag-derived specimens. In total, we analyzed 1244 pools. During field work, ticks were stored in liquid nitrogen and subsequently transferred to −80°C freezer in the laboratory.

The sample preparation procedure involved a washing step, where ticks were first washed in 70% ethanol, followed by two washes in sterile saline (0.9% NaCl). Immediately after washing, homogenization was performed in sterile normal saline using steel beads and a TissueLyser II (Qiagen, Hilden, Germany). The resulting homogenate was then clarified by centrifugation at 3000 rpm for 2 min to remove debris.

Nucleic acid extraction was carried out using 100 µL of the homogenate with the RiboPrep kit (Amplisens, Moscow, Russia), strictly following the manufacturer’s protocol.

### 2.2. PCR and Sequencing

Tick species identification was confirmed through sequencing of a cytochrome c oxidase I (COI) gene fragment using previously published primers [32], as well as by sequencing of the 16S rRNA gene fragment with primers 16+1 and 16-1 according to Black and Piesman [33]. For confirmatory sequencing, we used non-pooled samples (one tick per sample). We attempted to select specimens from each detected tick species, prioritizing samples from different provinces when possible. For species represented by few specimens, all available samples were sequenced. PCR screening for the presence of *Borrelia* sp. genetic markers was conducted using qRT-PCR with genus-specific primers targeting the 16S rRNA gene (Bor-16s-F 5′-AGCCTTTAAAGCTTCGCTTGTAG-3′, Bor-16s-R 5′-GCCTCCCGTAGGAGTCTGG-3′ and probe 5′-CCGGCCTGAGAGGGTGAACGG-3′). For the *Borrelia*-positive pools, sequencing of a flagellin gene fragment was performed using primers according to Stromdahl et al. [34]. The detection of *C. burnetii* was performed using the Amplisens *Coxiella burnetii* commercial kits (Amplisens, Moscow, Russia) in accordance with the manufacturer’s protocols. Positive samples were sequenced using the following primers: Cox-F1 5′-AGCGAACCATTGGTATCGGACGTT-3′, CoxBurSeq-R2 5′- TCATTGGCTTTTGCCACCGCT-3′. During pathogen screening, appropriate internal and negative extraction controls, as well as positive and negative PCR controls, were used to prevent false-positive and false-negative results.

We cleaned amplicons using ExoSAP-IT™ Express PCR Product Cleanup Reagent (Thermo Fisher Scientific, Vilnius, Lithuania). We added 1–3 µL of PCR product and 1 µL of ExoSAP to the reaction, depending on estimated amplicon concentration from agarose gel band intensity. The mixture incubated at 37 °C for 15 min, followed by enzyme inactivation at 80 °C for 15 min. The resulting purified PCR products were subjected to bidirectional Sanger sequencing with a BigDye Terminator v1.1 Cycle Sequencing Kit (Thermo Fisher Scientific, Waltham, MA, USA) on an Applied Biosystems 3500xL Genetic Analyzer (Applied Biosystems, Thermo Fisher Scientific, Foster City, CA, USA). 

### 2.3. Data Analysis

Following Sanger sequencing, the raw sequence data were processed using the Geneious software package (Biomatters Ltd., Auckland, New Zealand) (version 2020.2.5). This processing involved contig assembly, error checking, and sequence alignment. Phylogenetic analysis was subsequently performed using the MEGA X software. The minimum infection rate (MIR) was used to estimate the infection rate in pooled tick samples [35].

### 2.4. Ethical Statement

Tick collection from domestic animals was conducted with the verbal informed consent of the animal owners. The study protocol and procedures for sample collection were reviewed and approved by the ethical committee for animal research at the joint Russia-Vietnam Tropical Science and Technology Research Center (approval numbers 3225/CN-HDDD and 2707/CN-H ƉƉƉƉV). All methods were performed in accordance with relevant guidelines and regulations. Permission for field collection in Cat Tien National Park and other locations was granted by the Department of Forestry and Forest Conservation National Park Cat Tien, (Permit No. 452/VCT-KHHTQT).

## 3. Results

### 3.1. Ixodid Tick Fauna Data

Ticks were collected from 241 domestic animals, comprising 44 buffaloes, 107 cattle, 88 dogs, and two cats. An additional 474 ticks were collected from vegetation, exclusively in Dong Nai Province within the Cat Tien National Park. In total, 2347 ixodid ticks were obtained (Table 1).

Our sample encompassed ticks of eight species from three genera: *Haemaphysalis*, *Rhipicephalus*, and *Amblyomma*. An analysis of species prevalence revealed that the most abundant species was *R. microplus*, which dominated the collections from cattle and buffalo across all locations except for Dong Nai Province, where it was entirely absent from our sample. *R. microplus* was also found parasitizing dogs in the July and August collections. For ticks of the genus *Haemaphysalis*, we identified differences in the prevalence of various species between the northern and southern provinces. Specifically, in the northern provinces of Yen Bai and Cao Bang, the most prevalent *Haemaphysalis* species was *H. cornigera*; *H. bispinosa* was found infrequently, while other representatives of the genus were absent from the sample. In collections from the southern and central provinces of Dong Nai and Quang Ngai, the most prevalent *Haemaphysalis* species were *H. bispinosa* and *H. lagrangei*, with *H. hystricus* and *H. wellingtoni* detected infrequently. The dominant ectoparasite on dogs across all provinces was *Rhipicephalus linnaei*, which was also found on cattle, buffalo, and even cats. Representatives of the genus *Amblyomma* were encountered only a single nymph, collected from a cow in Yen Bai Province.

**Figure 1:**
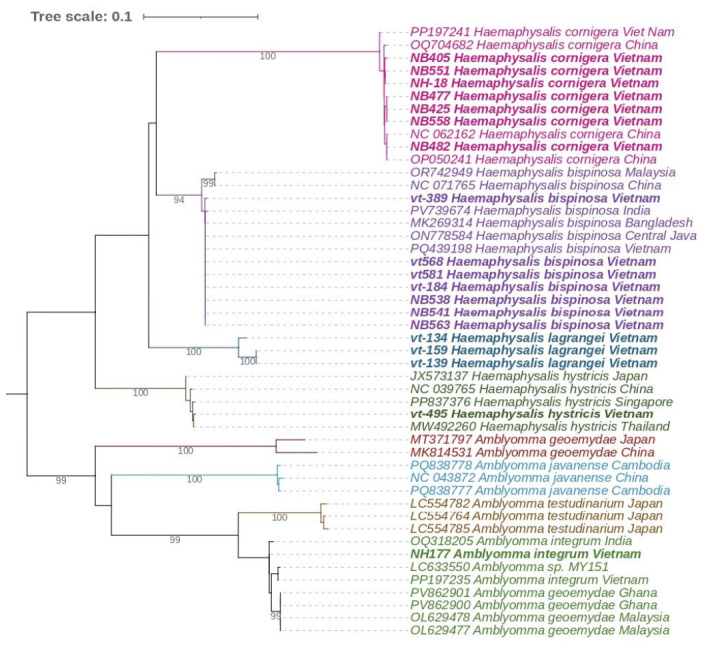
Phylogenetic tree based on an alignment of a 530 bp fragment of the cytochrome c oxidase I (COI) gene from ixodid ticks. The tree was constructed using the Maximum Likelihood method with the General Time Reversible model. Bootstrap support was calculated from 1000 replicates; branches with support values greater than 90% are indicated. Sequences obtained in the present study are highlighted in bold.

**Figure 2:**
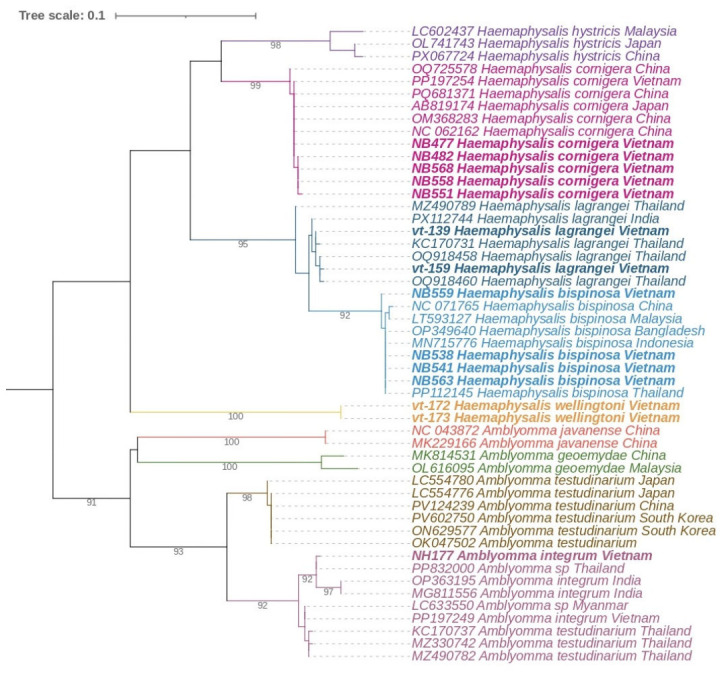
Phylogenetic tree based on an alignment of a 403 bp fragment of the 16S rRNA gene from ixodid ticks. The tree was constructed using the Maximum Likelihood method with the Tamura 3 parameter model. Bootstrap support was calculated from 1000 replicates; branches with support values greater than 90% are indicated. Sequences obtained in the present study are highlighted in bold.

For each of the eight tick species in our collection, we selected voucher specimens to confirm species identification by sequencing two mitochondrial targets. A total of 24 COI gene fragment sequences (GenBank accession numbers: PX701582–PX701595, PZ135096– PZ135105) and 15 16S rRNA gene fragment sequences (GenBank accession numbers: PX715045–PX715048, PZ135115–PZ135125) were obtained. This list includes sequences from an *Amblyomma* nymph collected from a cow in Yen Bai Province. The nymph was damaged during collection, preventing reliable morphological species identification. Sequencing of both COI and 16S rRNA gene fragments indicated that our specimen belongs to the species *A. integrum* (Figure 1 and Figure 2).

### 3.2. Molecular Screening and Genetic Characterization of Borrelia sp.

A total of 2327 ticks (1222 pools) were screened for the presence of *Borrelia* spp. RNA/DNA using qRT-PCR. Twenty-eight positive pools were identified, with the detection frequency varying by tick species and region (Table 2). The highest number of positive pools were detected in *H. cornigera* ticks collected from cattle and buffalo in Cao Bang Province, with a minimum infection rate (MIR) of 7.8%. In Yen Bai and Quang Ngai provinces, borreliae was detected only in *R. microplus*, showing a low MIR ranging from 1.1% to 2.2%. In Dong Nai Province, borreliae were found exclusively in larval ticks of *H. lagrangei* collected from vegetation in Cat Tien National Park. This collection site was inaccessible to domestic animals, indicating that these ticks feed on wild hosts such as muntjacs (*Muntiacus vaginalis*), sambar deer (*Rusa unicolor*), and gaurs (*Bos gaurus*). Notably, despite a substantial sample size, borreliae were not detected in *H. bispinosa* or *R. linnaei*.

**Figure 3:**
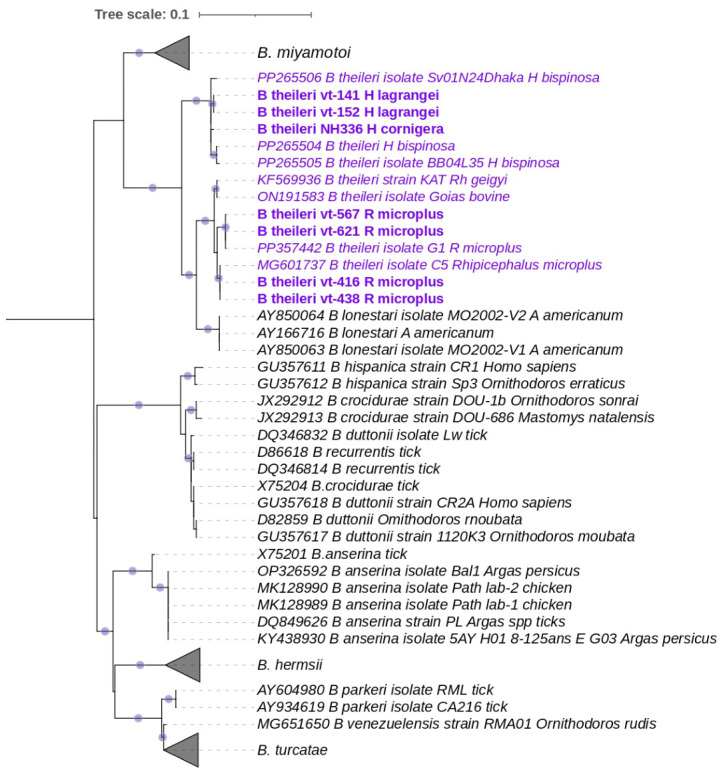
Phylogenetic tree based on an alignment of a 468 bp fragment of the flaB gene from *Borrelia* spp. The tree was constructed using the Maximum Likelihood method with the Tamura 3-parameter model. Bootstrap support was calculated from 1000 replicates; branches with support values greater than 80% are indicated by purple circular markers. Sequences obtained in the present study are highlighted in bold. The purple font color represents *B. theileri* sequences.

In the majority of positive pools, *Borrelia* spp. DNA was detected in low concentrations, with a median Ct-value of 32.0 (min = 17.0, max = 36.0). For eight pools with the highest DNA concentrations, sequences a fragment of the flagellin gene (flaB) were successfully obtained. Phylogenetic analysis revealed that the detected *Borrelia*-positive specimens belong to the relapsing fever *Borrelia* group and are most closely related to *Borrelia theileri* (Figure 3). On the phylogenetic tree, *B. theileri* sequences form two distinct groups separated from each other. One group includes *B. theileri* sequences obtained from ticks of the genus *Haemaphysalis*, including our isolates from *H. cornigera* and *H. lagrangei*. The second group includes *B. theileri* sequences obtained from ticks of the subgenus *Boophilus*, including our isolates from *R. microplus* (Figure 3). The phylogenetic distances between these two groups of *B. theileri* are comparable to those observed between other *Borrelia* species (e.g., *B. theileri* vs. *B. lonestari*, *B. hispanica* vs. *B. crocidurae* vs. *B. duttoni*) (Figure 3). 

### 3.3. Molecular Screening for Coxiella burnetii

A total of 2327 ticks (1222 pools) were screened for the presence of *C. burnetii* DNA using qPCR, of which 13 pools yielded a positive result. The majority of positive pools contained the target DNA at low concentrations, with a median Ct-value of 29.9 (min = 10.78, max = 35.53). For three pools with the highest DNA concentrations, sequences a fragment of the IS1111 element were successfully obtained (GenBank accession numbers: PZ268203-PZ268205). BLAST analysis (Web version BLAST NCBI) of these sequences showed 100% identity with *C. burnetii* sequences from humans (e.g., strains Schperling CP014563, Heizberg CP014561, and others), from goats (e.g., strains Namibia CP007555, Yunnan-1 KR697576, and others), and from ixodid ticks (PX422556, PV102519, PV102515, OP046711). The distribution of the examined ticks by species and region was the same as for the *Borrelia* spp. screening (see Table 2). The analysis revealed that all infected ticks were collected from domestic animals in Cao Bang Province. The positive specimens comprised 10 out of 197 *H. cornigera* (MIR = 5.1%) and 3 out of 95 *R. microplus* (MIR = 3.2%). Infected ticks were collected from cattle at four locations, corresponding to eight households within Phan Thanh Commune, Nguyen Binh District. These households are situated in close proximity to one another and share common grazing areas for their livestock. The spatial clustering of positive samples, combined with detection of *Coxiella* in only this province, points to localized circulation.

## 4. Discussion

The present study contributes to the understanding of ixodid tick distribution in Southeast Asia. A significant finding is the identification of a widespread prevalence of *H. bispinosa* in the southern and central provinces of Vietnam. This species was not previously included in local ixodid faunal records, with its presence in Vietnam first documented only in 2025 based on four females, two males collected from a dog, and one nymph from vegetation in Cuc Phuong Commune, Nho Quan District, Ninh Binh Province [12]. In our collections, this species was found in Cao Bang, Dong Nai and Quang Ngai provinces, parasitizing cattle, dogs, and even cats (Table 1). Notably, in Dong Nai Province, this tick species was the most abundant in collections from domestic animals. This result substantially expands the initial 2025 record of *H. bispinosa* in Vietnam. Our data indicate that *H. bispinosa* is more widely distributed within the country, necessitating further research in other provinces to map its full range in Vietnam. This tick species is widely distributed across South and Southeast Asia, with a range extending from Pakistan in the west to Indonesia, Thailand, and China in the east [36,37,38]. *Haemaphysalis bispinosa* parasitizes domestic ungulates (cattle, goats, sheep), dogs, and also birds [37], a broad host tropism that may facilitate its dispersal into new territories. The medical and veterinary significance of this species remains insufficiently studied and requires further investigation.

The detection of *H. cornigera* in Yen Bai and Cao Bang provinces also contributes to the knowledge of Vietnam’s ixodid fauna. This species was first reported in Vietnam in 2022 from two ticks in Son La Province [11], which, at the time of our study, constituted the sole record of the species in the country. Our work confirms the presence of *H. cornigera* in the northern provinces of Yen Bai and Cao Bang, while the species was absent from our samples from southern provinces. This identification was confirmed through selective sequencing.

Another noteworthy finding was the detection of an *A. integrum* nymph on a cow in Yen Bai Province, with species identification confirmed through sequencing of COI and 16S rRNA gene fragments. It is important to mention that the sequences available in GenBank that are phylogenetically close to our specimen include annotations for *A. integrum*, *A. geoemydae*, and even *A. testudinarium* (see, for example, accession numbers MZ490782, MZ330742, and KC170737). However, upon closer examination of other available sequences, it is evident that *A. testudinarium* forms a distinct, well-separated group based on both COI and 16S rRNA genes. Consequently, the samples that clustered together with *A. geoemydae* and *A. integrum* were most likely misidentified. A similar situation exists for *A. geoemydae*: GenBank contains two mitochondrial sequences from *A. geoemydae* from China and Japan (accession numbers MK814531 and MT371797, respectively), which form a separate clade in phylogenetic trees based on both COI and 16S rRNA genes. This clade is sufficiently distinct from other members of the genus *Amblyomma*, as expected for an independent species. Conversely, sequences from Malaysia, Thailand, and Ghana, deposited in GenBank as *A. geoemydae* (Figure 1) but phylogenetically close to *A. integrum* sequences, are also likely misidentified and belong to the species *A. integrum*. Support for this conclusion comes from sequences of *A. integrum* obtained in Vietnam in 2024 [11]. Their work included not only sequencing but also morphological comparison, confirming that the *Amblyomma* they found belonged to *A. integrum*. Additional supporting evidence is the fact that our *Amblyomma* nymph was collected from a cow, whereas *A. geoemydae* is known to parasitize turtles, unlike *A. integrum*, which parasitizes cattle. Therefore, based on the phylogenetic analysis of two genetic markers, we can conclude that the nymph we found belongs to the species *A. integrum*. This species was also first recorded in Vietnam only in 2024, with a single female collected from a cow in Son La province [11]. Our finding from another northern province of Vietnam corroborates this initial record and provides further evidence that its presence is not a sporadic event, allowing its inclusion in the list of Vietnam’s tick fauna.

Our research on tick-associated infections was focused on the main bacterial pathogens—*Borrelia* and *Coxiella*. Our results showed the presence of *B. theileri* in ticks, which is the first molecular confirmation of the presence of this pathogen in Vietnam. This spirochete belongs to the relapsing fever *Borrelia* group and is associated with ixodid ticks. *Borrelia theileri* is widely distributed in the world [39]; in particular, its presence in Asia is confirmed for South Korea, China, Thailand and Malaysia [40,41,42,43].

*Borrelia theileri* is not pathogenic for humans, but causes bovine borreliosis in livestock including cows, goats, and sheep [39]. This veterinary disease usually proceeds in a relatively mild form, causing fever, loss of appetite, anemia, hemoglobinuria, and reduced milk production. However, such a course of the disease, although not leading to the death of animals, nevertheless entails economic losses for farmers when widely distributed among the livestock. Our finding of *B. theileri* in Vietnam indicates the circulation of this pathogen in all four studied provinces and consequently raises the question of the prevalence of this pathogen in other regions of the country. To assess the significance of *B. theileri* for animal husbandry in Vietnam, additional studies of both ticks and animals with symptoms similar to bovine borreliosis are needed.

Analysis of the *flaB* gene fragment revealed two distinct clusters of *B. theileri* sequences: one associated with *Haemaphysalis*, and the other with *Boophilus*. The genetic distance between these clusters was similar to that seen between separate relapsing fever *Borrelia* species, indicating significant divergence between the two groups. However, the *flaB* gene fragment alone does not provide enough information for detailed analysis, and comparisons based on other genes or whole genomes are needed.

Detection of *Coxiella* DNA in 13 ticks from Cao Bang Province was performed using a commercial PCR kit. This kit is recommended by the manufacturer for screening biological materials from humans (whole blood, sputum, bronchial lavage fluid, cerebrospinal fluid, autopsy material), from animals (blood, placenta, aborted fetal material, autopsy material), and for ixodid ticks. The manufacturer states that the specificity of this kit has been validated and that there is no cross-reactivity with phylogenetically related bacterial genera (*Rickettsia, Ehrlichia, Francisella*). We obtained sequences a fragment of the IS1111 element from three positive samples. BLAST analysis showed 100% identity of these sequences with *C. burnetii* sequences. However, it was not possible to compare them with sequences of *Coxiella*-like endosymbionts because no a fragment of the IS1111 element sequences for these bacteria are available in GenBank. Therefore, at this stage of the study, we can only conclude that bacteria of the genus *Coxiella* were detected. Nevertheless, it is worth noting that if this PCR kit also detected *Coxiella*-like endosymbionts of ticks, the number of positive ticks would likely be higher than 13 (out of a total of 1222 pools). Furthermore, such samples would probably not be spatially clustered in a single location but would be more widely distributed across the study sites. These observations provide indirect evidence supporting the detection of *C. burnetii*, but additional sequences are needed for unambiguous confirmation.

*Coxiella burnetii*, the causative agent of Q fever, is a significant bacterial zoonosis with a global distribution. This obligate intracellular pathogen infects a wide range of animals, with ruminants serving as the primary reservoir [44]. Transmission occurs predominantly via aerosolized particles, especially during parturition, as the bacterium exhibits a high tropism for placental tissues [45]. While often asymptomatic in livestock, infection can lead to reproductive losses and the shedding of bacteria in milk, posing substantial risks to both animal productivity and public health [46]. The epidemiological role of ticks is also recognized, as *C. burnetii* can replicate within and be shed through tick faeces, facilitating environmental contamination and transmission [47,48].

Data on *C. burnetii* in Southeast Asia remain limited, but serological evidence confirms its circulation in the region [44]. Studies have reported seroprevalence in goats in Cambodia (7.2%) and Thailand (3.5%), and in cattle in Lao PDR (3–4%) and Thailand (4.6–6%) [49,50,51,52,53,54,55]. The human health impact is underscored by seropositivity rates of 12.6% among Thai individuals and confirmed human cases [56], including a recent molecularly confirmed infection in a man from Hai Phong, Vietnam, in 2022 [30,57]. This case provides definitive evidence of the pathogen’s presence in the country and underscores the necessity for enhanced surveillance. Our detection of *Coxiella* DNA in ticks collected from domestic animals requires further investigation, including definitive species identification, assessment of prevalence, and evaluation of the potential role of ticks in the local transmission cycle of Q fever.

## 5. Conclusions

In summary, our study contributes to the knowledge of the distribution of ixodid ticks in Vietnam. We provide new data on the occurrence of *H. bispinosa*, *H. lagrangei*, *H. wellingtoni*, *H. hystricus*, and *A. integrum* in the country, a finding is important for understanding the epidemiology of tick-borne diseases in the region.

Our results demonstrate that *Borrelia* spp. DNA was present in ticks across all studied regions of Vietnam. The overall infection rate was relatively low, with a notable concentration in *H. cornigera*. Selective sequencing enabled the identification of *B. theileri*, the causative agent of bovine borreliosis.

Furthermore, we found evidence of *Coxiella* circulation in ticks parasitizing domestic livestock in Cao Bang Province. Further studies of both ticks and samples from domestic livestock in this province are needed to confirm or rule out the circulation of *C. burnetii* at the tick-livestock interface.

## Figures and Tables

**Table 1:** Collection data on Ixodid tick fauna from various hosts and from vegetation in selected provinces of Vietnam.

Tick Species/Host	Number of Ticks				
	Yen Bai	Cao Bang	Dong Nai	Quang Ngai	Total
Host: Cattle, studied animals *	23	50	40	38	151
*Haemaphysalis cornigera*	73	198	0	0	271
*Haemaphysalis bispinosa*	0	4	257	21	282
*Haemaphysalis* sp.	7	0	0	0	7
*Rhipicephalus microplus*	181	95	0	278	554
*Rhipicephalus linnaei*	0	0	14	6	20
*Amblyomma* sp.	1	0	0	0	1
Host: Dogs, studied animals	1	0	34	53	88
*Haemaphysalis cornigera*	3	**	0	0	3
*Haemaphysalis bispinosa*	0	–	44	29	73
*Haemaphysalis hystricus*	0	–	0	2	2
*Rhipicephalus microplus*	0	–	0	135	135
*Rhipicephalus linnaei*	24	–	163	312	499
Host: Cats, studied animals	0	0	0	2	2
*Haemaphysalis bispinosa*	–	–	–	14	14
*Rhipicephalus linnaei*	–	–	–	9	9
Source: Vegetation					
*Haemaphysalis lagrangei*	0	0	474	0	474
*Haemaphysalis wellingtoni*	0	0	3	0	3
Total	289	297	955	806	2347

*—Number of host animals examined. **—host animal not sampled in this region; tick data absent.

**Table 2:** Detection of *Borrelia* spp. in tick pools: number of positive pools and Minimum Infection Rate (MIR).

Tick Species	Yen Bai		Cao Bang		Dong Nai		Quang Ngai	
	N *	PCR+ **	N	PCR+	N	PCR+	N	PCR+
*H. bispinosa*	0	0	4	0	301	0	64	0
*H. cornigera*	75	0	185	14 (7.6%)	0	0	0	0
*H. hystricus*	0	0	0	0	0	0	2	0
*H. wellingtoni*	0	0	0	0	3	0	0	0
*H. lagrangei*	0	0	0	0	474	2 (0.4%)	0	0
*R. microplus*	180	2 (1.1%)	88	1 (1.1%)	0	0	413	9 (2.2%)
*R. linnaei*	24	0	0	0	177	0	330	0
*A. integrum*	1	0	0	0	0	0	0	0

* N is the total number of studied ticks. ** PCR+ is the number of PCR-positive pools (MIR%).

## Data Availability

The data that support the findings of this study are publicly available from GenBank with the identifiers PX701582–PX701595, PZ135096–PZ135105, PX715045–PX715048, PZ135115–PZ135125, PX714834–PX714837, and PZ268203-PZ268205.
